# Pneumatose kystique intestinale révélée par une sténose d'une anastomose gastro-jéjunale: à propos d'un cas

**DOI:** 10.11604/pamj.2013.15.125.2727

**Published:** 2013-08-08

**Authors:** Mohammed Amine El Matiallah, Hicham El Bouhaddouti, Ouadii Mouaqit, El Bachir Benjelloun, Abdelmalek Ousadden, Khalid Ait Taleb

**Affiliations:** 1 Service de Chirurgie Viscérale A, CHU Hassan II, Fes, Maroc

**Keywords:** Pneumatose kystique intestinale, sténose, anastomose gastro jéjunale, gastrectomie, Cystic pneumatosis intestinal, stenosis, gastro jejunal anastomosis, gastrectomy

## Abstract

La pneumatose kystique intestinale est la présence de bulles gazeuses dans la paroi et les séreuses du tube digestif. Il s'agit d'une pathologie bénigne, rare, de diagnostic radiologique et de traitement médical. Nous rapportons le cas d'un homme âgé de 42ans, opéré il y a 6ans pour une sténose du bulbe duodénal d'origine ulcéreuse, il avait bénéficié d'une gastro-entéro-anastomose avec bivagotomie tronculaire. Il a été hospitalisé pour des vomissements associés à des épigastralgies. le patient a bénéficié d'une fibroscopie oeso-gastro-duodénale qui a trouvé une stase gastrique gênant toute exploration, ce qui a conduit à la réalisation d'une tomodensitométrie abdominale qui a objectivé un énorme estomac de stase en amont d'une sténose de l'anastomose gastro jéjunale, une pneumatose kystique intestinale et un pneumopéritoine. Le patient a été opéré et l'exploration a trouvé une ascite, un volumineux estomac de stase et des adhérences entre le grêle et le colon droit, sièges de la pneumatose, provoquant un tour de spire (volvulus) de l'ancienne anastomose gastro-jéjunale. L'estomac était atone. Une gastrectomie des 2/3 emportant l'ancienne anastomose suivie d'une anastomose type Finsterer manuelle a été réalisée. Les suites post opératoires étaient simples. La pneumatose kystique intestinale est une affection bénigne, de diagnostic radiologique. Le scanner permet d’étudier la diffusion des gaz dans les séreuses digestives. Son traitement est habituellement médical alors que ses complications peuvent relever d'un traitement chirurgical comme pour notre patient.

## Introduction

La pneumatose kystique intestinale est la présence de bulles gazeuses dans la paroi et les séreuses du tube digestif. Il s'agit d'une pathologie bénigne, rare, pauci-symptomatique, de diagnostic radiologique et de traitement médical. On distingue classiquement une forme primitive ou idiopathique (15% des cas) et une forme secondaire (85% des cas) aux associations pathologiques nombreuses, de traitement étiologique [1–2].

## Patient et observation

**Figure 1:**
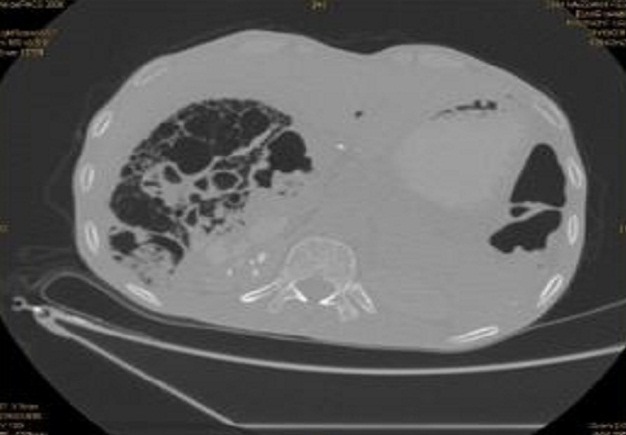
Tomodensitométrie abdominale, coupe axiale montrant la pneumatose kystique intestinale

**Figure 2:**
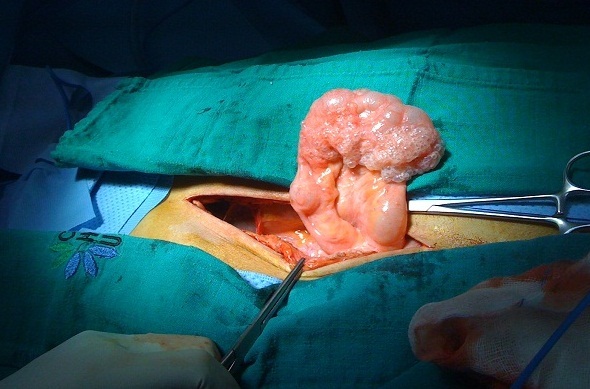
Image per opératoire montrant la pneumatose kystique intestinale

**Figure 3:**
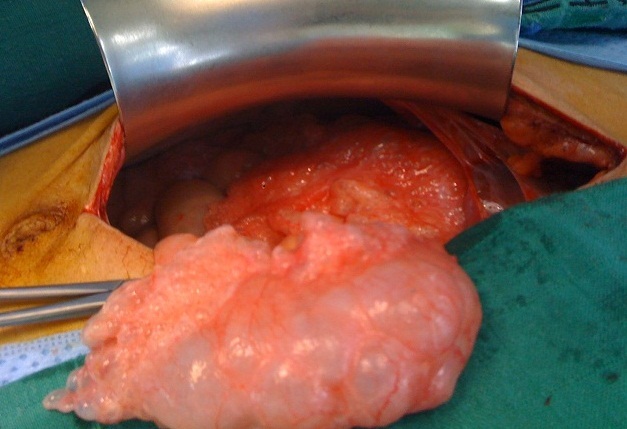
Image per opératoire montrant la pneumatose kystique intestinale

Monsieur M.H, âgé de 42ans, tabagique chronique depuis 20ans, suivi pour BPCO depuis 5ans et opéré il y a 6ans pour une sténose du bulbe dudodénal d'origine ulcéreuse, il avait bénéficié d'une gastro-entéro-anastomose avec bivagotomie tronculaire. Il a été admis aux urgences pour des vomissements post-prandiaux tardifs évoluant depuis une semaine associés à des douleurs épigastriques sans autre signes accompagnateurs. L'examen clinique a trouvé un patient apyrétique, un clapottage à jeun et des signes de déshydratation. Le bilan biologique a montré une hypokaliémie à 2.7mEq/l, une hypoalbuminémie à 26g/l, l'hémoglobine était à 11g/dl et la leucocytose à 11.000 éléments/mm3. Devant ce tableau, le patient a bénéficié d'une FOGD qui a trouvé une stase gastrique importante gênant toute exploration malgré les tentatives d'aspiration, ce qui a conduit à la réalisation d'une tomodensitométrie (TDM) abdominale qui a mis en évidence une pneumatose kystique au dépend d'une paroi digestive avec un pneumopéritoine et une disposition anormale des anses grêles au niveau du flanc droit (Figure 1). Il n'a pas été noté de thrombose vasculaire mésentérique ni d'aéroportie. Le patient a été opéré par voie médiane itérative, et l'exploration a trouvé une ascite d'environ 500cc, un estomac de stase très volumineux secondaire à des adhérences entre le grêle et le colon droit, sièges de la pneumatose, provoquant un tour de spire (volvulus) de l'ancienne anastomose gastro-jéjunale (Figure 2, Figure 3). L'estomac était très peu péristaltique, donc une gastrectomie des 2/3 emportant l'ancienne anastomose suivie d'une anastomose type Finsterer manuelle a été réalisée. Les suites post opératoires étaient simples.

## Discussion

La pneumatose kystique intestinale se définit comme la présence de kystes gazeux dans la paroi digestive pouvant siéger de l’oesophage au rectum, mais préférentiellement sur le grêle et le côlon gauche [1–3]. Il s'agit d'une pathologie bénigne, peu fréquente, à prépondérance masculine après 50 ans [4] et majoritairement secondaire, décrite en association avec diverses affections (Tableau 1).

**Table 1:** **Affections** associées à la pneumatose kystique intestinale

**Maladies de système**
Polyartérite noueuse
Lupus érythémateux disséminé
**Affections digestives**
Traumatisme abdominal
Maladies inflammatoires de l'intestin
Sténose pylorique, ulcère gastro-duodénal
Anastomoses intestinales, grêle court, court-circuit jéjuno-iléal
Entéropathie diabétique
Maladie cæliaque
Maladie de Hirschsprung
**Autres**
Gestes invasifs
Colonoscopie
Médicaments
AINS, corticoïdes, ciclosporine, neuroleptiques
BPCO, asthme et Cardiopathies
Toxiques
Trichloro-éthylène
Infections
CMV, HIV, Clostridium difficile, autres.

Les kystes affectent l'intestin grêle dans 42% des cas, le colon dans 36%, et plus rarement l’oesophage et le rectum [5]. La majorité des pneumatoses primaires est limitée au colon plus particulièrement au sigmoïde et au colon gauche; celle touchant l'intestin grêle est plutôt secondaire [2]. Du point de vue anatomopathologique les kystes mesurent de quelques millimètres à plusieurs centimètres, présentent un aspect polypoïde bleuâtre et se situent préférentiellement au niveau de la sous-muqueuse pour le colon et la sous-séreuse pour le grêle. Lorsque les kystes vieillissent, ils s'entourent de fibrose et des cellules géantes comblant leurs lumières.

La multiplicité des affections associées rend compte de la difficulté d’établir une théorie uniciste quant à sa physiopathogénie. La théorie mécanique avance l'hypothèse d'une pénétration des gaz digestifs sous l'effet d'hyperpression intraluminale à travers les tuniques de la paroi, favorisée par une muqueuse fragile ulcérée et inflammatoire [6]. Cette théorie s'appuie sur des observations anciennes de pneumatose associée à des ulcères digestifs, comme dans le cas de notre patient, sur des cas plus récents de pneumatose post-endoscopie digestive [4], et sur des observations de pneumatose contemporaines d'affections modifiant la motilité intestinale. L'effort de toux responsable d'hyperpression abdominale semble être un facteur favorisant la diffusion gazeuse. La théorie pulmonaire se rapproche de la théorie mécanique [6]. L'hyperpression thoracique chez les BPCO et les asthmatiques serait le point de départ d'une diffusion gazeuse, via le médiastin, vers les séreuses digestives suivant un trajet péri-vasculaire, péri-lymphatique et au sein des méso [3, 4, 7]. Enfin, la théorie bactérienne s'appuie sur l'analyse des bulles gazeuses dont la forte teneur en hydrogène et en azote, résultat de la fermentation bactérienne, s'explique par une pullulation microbienne de germes anaérobies dans la sous muqueuse (Clostridium perfringens, Enterobacter) [8]. La bonne réponse des pneumatoses coliques au métronidazole est en faveur de cette théorie. L'hypoxie chronique des BPCO serait un facteur favorisant le déséquilibre de la flore intestinale avec hyper production de gaz issu de la fermentation des hydrates de carbone. Ainsi, les deux théories seraient intriquées. Comme le propose Gigliardi: «les facteurs mécaniques d'hyperpression intra thoracique et abdominale initieraient la présence de gaz, entretenue par la suite par l'hyperproduction bactérienne gazeuse» [4]. Notre observation résume ces différentes théories. En somme, cette pneumatose a certainement une double origine: digestive en raison de l'ulcère bulbaire, et thoracique, chez ce patient atteint de BPCO.

La symptomatologie clinique de la pneumatose intestinale est habituellement aspécifique et généralement celle de la maladie causale. Vomissements post-prandiaux, déshydratation, perte de poids et douleurs abdominales) [1, 7–11]. Cette absence de spécificité rend l'imagerie d'un intérêt majeur pour le diagnostic positif et étiologique. L'ASP montre des hyperclartés kystiques en « grappe de raisin» adjacentes à la lumière digestive réalisant un aspect de double contour gazeux évocateur du diagnostic [12]. Le scanner multi-détecteurs est très performant pour mettre en évidence l'aspect perlé ou la pariétographie gazeuse [13]. L'utilisation de fenêtres larges pulmonaires est indispensable. Le scanner permet en outre de préciser l’étendue des lésions pathologiques, de suivre la diffusion anatomique du fluide gazeux, de préciser le caractère primitif ou secondaire de l'affection et surtout de distinguer la pneumatose kystique de l'ischémie mésentérique aiguë avec pneumatose pariétale et recherchera des signes de complications tels que la perforation, l'hémorragie ou l'occlusion [14], comme ce qui a été trouvé chez notre patient. Le pneumopéritoine et le rétro-pneumopéritoine sont l'apanage de la rupture des kystes sous séreux [15].

La pneumatose kystique primitive évolue habituellement de façon spontanément favorable sans traitement [16] et dans les formes secondaires par un traitement associant un régime sans résidus, une antibiothérapie par métronidazole (Flagyl^®^) destinée à lutter contre la production gazeuse bactérienne et, pour certains, une oxygénothérapie hyperbare [17, 18]. L'inefficacité du traitement, la nature de la pathologie causale ou la survenue de complications modifiera la prise en charge thérapeutique qui deviendra dès lors chirurgicale [19]. Ce qui est le cas dans notre observation où l'intervention chirurgicale s'est imposée en raison de la sténose de l'anastomose gastro-jéjunale.

## Conclusion

La pneumatose kystique du grêle est une affection bénigne peu connue, de diagnostic radiologique. Elle ne doit pas être confondue avec un simple pneumopéritoine, ni une pneumatose sur gangrène intestinale. Pour cela, le scanner multi détecteur est extrêmement performant pour étudier la diffusion des gaz dans les séreuses digestives, les différents espaces péritonéaux, pré péritonéaux et rétro péritonéaux. Il guide le chirurgien pour traiter une éventuelle complication, en l'occurrence une sténose de l'anastomose gastro-jéjunale.
